# Creating a Social Learning Environment for and by Older Adults in the Use and Adoption of Smartphone Technology to Age in Place

**DOI:** 10.3389/fpubh.2021.568822

**Published:** 2021-06-16

**Authors:** Marjolein den Haan, Rens Brankaert, Gail Kenning, Yuan Lu

**Affiliations:** ^1^Department of Industrial Design, Eindhoven University of Technology, Eindhoven, Netherlands; ^2^Institute of Allied Health Professions, Fontys University of Applied Sciences, Eindhoven, Netherlands; ^3^Ageing Futures Institute, University of New South Wales, Sydney, NSW, Australia; ^4^Faculty of Arts and Social Sciences, University of Technology Sydney, Sydney, NSW, Australia

**Keywords:** aging in place, healthy aging, older adults, learning, smartphones

## Abstract

Smartphone technologies can support older adults in their daily lives as they age in place at home. However, they may struggle to use these technologies which impacts acceptance, adoption, and sustainable use. Peer to peer community learning has the potential to support older adults to learn using (smartphone) technologies. This paper studies such a learning community approach and how it can support older adults to learn using and adopt the smartphone application GoLivePhone. This technology assists older adults in their daily living by supporting them through fall detection and activity tracking. In particular, the interface of this application can evolve and adapt as older adults become more knowledgeable during the use process or as their abilities change. This paper shows a field study with seven older adults learning and using the GoLivePhone technology through a living lab approach. These older adults participated in this research in a technology learning community that was set-up for research purposes. For this we used ordinary Samsung A3 smartphones with the simplified GoLivePhone software, particularly designed for older adults. At the end of the learning class we conducted an additional focus group to both explore factors facilitating older adults to learn using this technology and to identify their main personal drivers and motivators to start and adopt this technology. We collected qualitative data via open questions and audio recording during the focus group. This collected data was subject to a thematic analysis, coding was primarily performed by the first author, and reviewed by the other authors. We provide insights into how peer to peer community learning can contribute, and found both *super-users* and recall tools to be helpful to support sustainable use of smartphone technology to support older adults to age in place.

## Introduction

In this paper we align with the concept of healthy aging as being health beyond illness, and also consider enrichment, fun, and good quality of life. As per the World Health Organization guidelines of 2020: “Health is a state of complete physical, mental and social well-being and not merely the absence of disease or infirmity” (1).

Many older adults want to remain at home as they age, if possible, which means the individual's home needs to support continuity in the living environment and the maintenance of daily independence and social contact (2). As the needs of people living independently increase and extends beyond personal care, and with the number of older adults increasing, this potentially places a financial burden on the system (1). Therefore, effective, efficient ways of supporting older adults to remain independent are needed.

People aspiring to age in place can benefit from digital opportunities. But, how and why people use and adopt technology varies between older adults, and *in situ* research about aging in place is limited (3). Wang et al. (4) investigated the barriers and facilitators for adopting aging in place technologies in the United States (U.S.) population over 65 years of age. They found five factors impacting use: (1) technology usability, (2) technology literacy, (3) data management, (4) privacy attitudes, and (5) co-design. They recommended educating not only the older adults in the use of technology but also technology designers in the design.

Currently, society continues to enjoy many digital developments, such as technologies that promote exercise (5), prevent falls (6), and facilitate cognitive training (7). Furthermore, Information and Communication Technologies (ICT) are used for staying socially connected, accessing instant information, and performing everyday tasks such as shopping, traveling, and banking (8). The value of the newly introduced technology should be clearly communicated to older adults so that they can recognize the potential usefulness and benefits (9). Meanwhile, designers need to understand how the user experience can go beyond functionality to also emotionally engage older adults (10).

Alongside communicating clearly about the values of a particular technology, we also recognize people have different needs, wants, and dreams (11) and widely varying abilities (12–14). This means, it is important to have technology that is able to cater for these individual differences or be adaptable to them (15).

Several studies have shown the challenges and opportunities of mobile health interventions. Joe and Demiris (16) argue that older adults are more likely to have a mobile phone than a desktop or laptop. Therefore, mobile phones seem an ideal technology platform to reach many older adults. Furthermore, Klasnja and Pratt (17) reviewed the body of work on mobile phone health applications and concluded that there were five intervention strategies for such applications: (1) tracking health information, (2) involving the healthcare team, (3) receiving support from your social environment, (4) increasing the health information accessibility, and (5) promoting entertainment. All of these could potentially support older adults to age in place. However, there remain challenges with regard to using mobile health technology for older adults, for example, Wildenbos et al. (18) cognition, physical ability, perception, and motivation to negatively impact using mobile technology. Other barriers include issues with familiarity, willingness to ask for help, trusting technology, privacy, and challenges in catering for physical and cognitive changes associated with aging (19). Additionally, another study found that tablets are currently too complex and recommend reducing available options on them (20). Furthermore, there is a need to ensure there is appropriate support matching the experiences of older adults with (self) supporting measures, tools and social networks (20–23), that the context for use is optimized (3), and that actions are performed along with peers to positively influence learning (24).

In this study we therefore apply a peer learning model as it provides older adults with an effective and rewarding learning environment (25). We used a specific peer learning model, called super-users, which will be addressed in the material and method section. In our work, we study a specific mobile technology, the GoLivePhone, via a Living Lab approach. In this we explore how new technology is used in the “real-life” and engage with people in-context (26). The Living Lab setup allows participants to become active contributors during the evaluation of technology (27).

Smartphone technologies can support older adults as they age in place in their homes. However, adoption of smartphone technology is often still challenging for older adults. This paper engages with a community of independent older adults aged between 66 and 86 from a predominantly rural area in the Netherlands, while they learn how to use the novel smartphone technology. During this smartphone learning class we investigated the participants' motivators and barriers to start and continue learning using the smartphone technology; to observe older adults and understand *how* they learned, what facilitated this learning and to provide insights to the smartphone company.

## The Study—Materials and Methods

We explored through the study (1) How can older adults be assisted in effectively learning to use a smartphone which supports their independence? (2) What drives older adults to begin and continue using a smartphone which supports them in aging in place?

In the following sections, we will elaborate on: (1) the use of peer-to-peer teaching and a learning class in a Living Lab approach, (2) the role of participants as *users* and *super-users*, (3) the specific smartphone technology used, and (4) how data was collected.

### The Use of Peer-to-Peer Teaching and a Learning Class in a Living Lab Approach

Over the course of a 13 week period, seven older adults met every Friday afternoon from 2 pm to 4 pm as part of a smartphone learning class (with four peer teachers). The atmosphere of the sessions was informal with the group sitting around a coffee table in a community center called “The Living Room.” The community center was close-by for all older adults, being in the city center of a village, so they could easily reach it. This contributed to the sustained attendance of the group. The room was equipped with a projector and projector screen, which the lead researcher used to introduce the research study to potential participants through a presentation. Members of the smartphone learning class were invited to take part in a series of focus groups over a period of 5 weeks (out of the 13 weeks class). The focus group methodology was used to follow users' progress as they learned how to use a smartphone (28). Based on existing studies using focus groups it was expected that data saturation would be reached within 5 weeks, and attending the full 13 weeks would not provide additional information (29). For the 5 weeks when the focus groups took place two researchers were present during the session, and particularly at the end of the session most of the interaction took place between researchers and participants. A predefined set of topics was developed for discussion to capture prevailing opinions about smartphone technologies and evaluate usage and general experience. Participant responses were written down by the participants themselves, and in the final session, additionally, a transcript of an audio recording was made. All written answers and the transcript were coded by the lead researcher and analyzed by all co-authors. This approach was selected as it could provide feedback that could contribute to innovating technology development and use through the involvement of participants in a real-life setting (30). It could also promote group interaction and so provide better insights into the experiences and opinions of the participants (31).

### The Role of Participants as Users and Super-Users

A call for attendees for the smartphone learning class was made by an older adult, who had previously been trained in using the technology (identified in the research as a super-user), through a local association for older adults and a local newspaper. Attendees of the class were offered an opportunity to become acquainted with a smartphone aimed at fostering longer independent living. The class objective was to educate the local community by using volunteers and working with the local municipality and the local older adults association, to improve the environment for aging. The research study participants were the attendees of these pre-arranged learning sessions who agreed to take part in the focus groups and to be observed by researchers. The number of participants in the learning experience and the research study was small to ensure personal feedback could be provided to everyone who participated and to be manageable for the super-users to teach effectively.

The research study was part of the European AAL project *ENSAFE* (32) which aimed to support effective prevention and self-care strategies for older adults to foster independent living. We were not required by the university to obtain formal approval through an ethics board, however general ethical procedures were followed to protect the participants. All participants in the research study signed a consent form agreeing to share their experiences which would be de-identified and analyzed anonymously. The participants were made aware of how to contact the researchers for concerns, their participation was voluntary, and they could withdraw at any point. To ensure the overall well-being of all participants, one older adult, who hosted the learning session as a so-called super-user, was in charge of communicating to the researcher any discomfort or health issues expressed by participants.

The study participant group consisted of seven older adults who wanted to learn to use the smartphone, referred to as “users” (Table 1). For the research study, this constituted a purposive sample providing information-rich, in context, qualitative data (33). This sample size is appropriate for findings that are not intended to be generalizable across populations but are transferable to context-specific populations.

**Table 1:** Background information of our seven participants (P).

**P**	**Living situation**	**Frequency of using technology**	**Perceived technology level**
1	Living independently	Daily	None
2	Living with partner	N/A	N/A
3	Living independently	Daily	Low
4	Living with partner	Daily	Low
5	Living with partner	Daily	None
6	Living independently	Daily	Low
7	Living with partner	Daily	Low

The hosts or facilitators of the learning sessions, were called super-users because of three main characteristics, they: (1) were experienced users of this particular smartphone, (2) have similar social-cognitive profiles to the participants, meaning a similar age range and similar ability, and (3) trained in providing expertise on the technology at hand. These super-users, like the general attendees (users) were invited to become participants in the research study, with their presence, activities and influences observed alongside the other participants. Along with introducing and teaching the system step-by-step, these super-users simplified the text and structure of a printed manual based on what the company of the smartphone technology provided on their website, enabling the users to continue practicing at home. This reflects the position of Mitzner et al. (9), who suggests a manual “may not be optimal because they contain tech jargon.”

The four super-users had been in a similar program before and were informed and educated about the particular smartphone prior to the sessions and could download and install software on a Samsung Galaxy A3 (2016) using a descriptive manual provided by the company. A 1 h follow-up session of questions was organized by the company.

### The Specific Smartphone Technology Used

**Figure 1:**
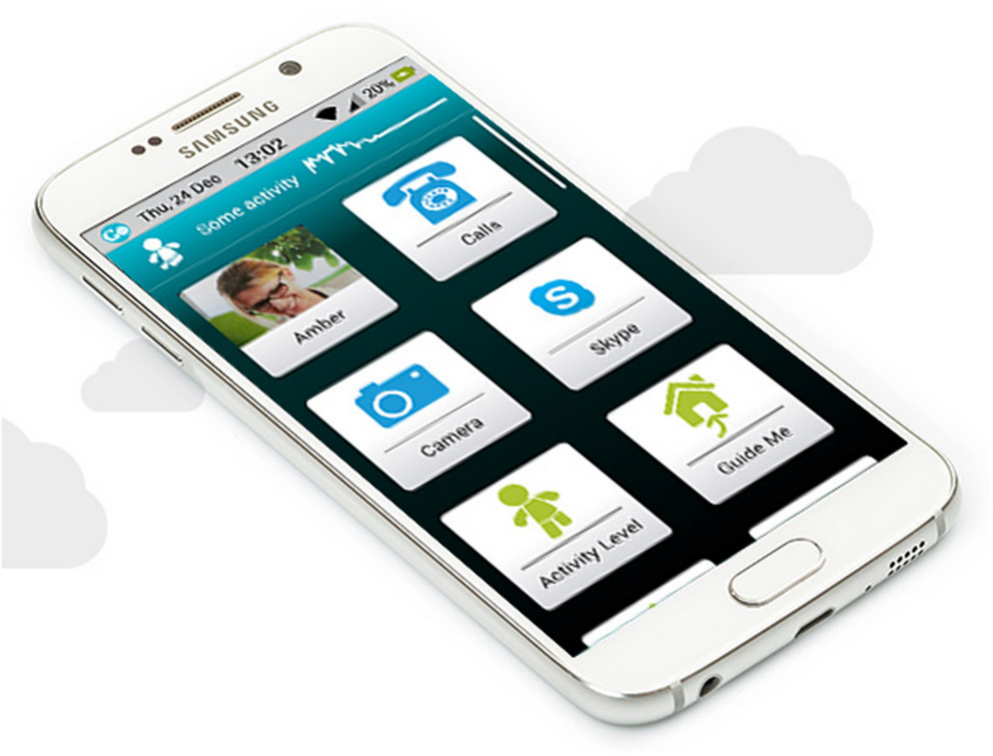
The Samsung smartphone with custom GoLivePhone User Interface, specifically designed for and evaluated with older adults (picture by Gociety Solutions in 2017).

The technology used in the learning class and research study was a smartphone Samsung A3 with a custom *GoLivePhone* user interface on “top” of the usual interface, explicitly designed for independently living older adults to age in place (Figure 1). “Independently living older adults” refers to older adults living with or without a partner in a regular home environment. The custom interface aims to make the interaction with the technology easier for older adults by offering clear pictograms, sizable icons, and high contrast. In addition to the common smartphone applications, this smartphone offers, amongst other things, fall prevention tips, fall detection, automatic activity tracking, and guidance to home or parking place (34). If desired, older adults can enable the sending of a warning to their (grand) children whenever a fall is detected or when a GPS zone is crossed (digital fencing), all aimed to create a digital remote support network to allow people to age in place. For the participants, keeping an overview on your health in this way was compared to taking your car for a regular check-up, showing how it could automatically track their activity by them simply carrying the smartphone in their pocket. Comparing their own health to car maintenance provided a metaphor to explain the concept of the technology and made users conscious about healthy aging as suggested by Mitzner et al. (9) when trying to clarify the potential benefit that technologies can bring.

The learning class introducing the smartphone focused on introducing three functionalities in the first session, to make the learning process manageable. These include connecting to Wi-Fi, managing contacts, and reaching out to somebody (either by calling or by using messaging service WhatsApp). In the second session, these functionalities were repeated, and three more functionalities were added, namely: using the camera, exploring photos via an album and sharing photos and videos using WhatsApp. All functionalities can be individually enabled or disabled in the main menu, in line with the older adult's interests, ability, and learning pace. An explanation of how to do this themselves was also given in the second session. To conclude, a group WhatsApp was created amongst participants for them to practice sharing photos and videos. In the third session, they repeated taking and sharing photos and videos. In addition, a new functionality was introduced to connect family members to their accounts, so they receive a notification if a fall occurs—if the user permits. In the fourth session, particular *GoLivePhone* applications were introduced, and in the final fifth session, a group discussion was done which was audio-recorded and the older adults were thanked for their participation in our research and given a postcard with a small present to thank them for their contribution in the study.

### How Data Was Collected

We held an open focus group after the learning class to let users reflect and voice their perspectives on the technology and learning process. This allowed older adults to actively participate and make their voices heard as equal partners in their introduction to, and assessment of the technology. The data were subject to a thematic analysis (35). This analysis was used to search for themes and patterns across the entire data set, rather than focusing on the responses of individual participants. By doing so, we found recurring use patterns for the whole group. The thematic analysis contained six phases, using the procedures described by Braun and Clarke (35): (1) familiarize yourself with the data by reading and noting down initial ideas, (2) generate initial codes across the entire data set, (3) search for potential themes by gathering codes, (4) review these themes and create a “map” of the analysis, (5) define and name each theme more to refine the specifics of each theme, and (6) produce the report on the final analysis with the selection of vivid, compelling extract examples.

## Results

Background information about the seven participants (P) is shown in Table 1, based on multiple-choice questions in which the frequency of using technology and “tech-savviness” of the participants were self-reported. For example, participants advised if they used desktop computer, phone (without internet), tablet, e-reader, smartphone, camera, smart television, technological care services or other technology. The only exclusion and inclusion criteria were that they need to be able to read the smartphone screen and be physically able to interact with it, and so in practice, this meant most of the participants had not used a smartphone before.

Through a process of familiarization with the focus group data, initial codes were generated, and searches for potential themes were carried out. The two main overarching themes were related to “learning” and “personal drivers,” each with multiple themes and subthemes (Table 2). “Learning,” related to how people prefer to learn, which tools contribute to learning, and who facilitates learning. “Personal drivers,” related to information about why people started using the phone and what keeps them motivated to continue doing so. We will provide more details on these themes and illustrate the content by including quotes from participants. As the researcher joined five of the sessions, we will phrase the specific quotes of participants (P) and super-users (SU) in time as Q1, Q2, Q3, Q4, and Q5, respectively.

**Table 2:** Our thematic analysis with the two overarching themes “learning” and “personal drivers” including their themes and subthemes.

	**Theme**	**Subthemes**
Learning	Step by step	In class guidance Introduction of tech options
	Repetition
	Tools	Manual Quick reference guide
	Who facilitates the learning
Personal drivers	Why start	Preparation for the future Move with the times
	Social
	Product-related values	Feeling of safety Accessibility

### Learning

Learning consisted of four themes: (1) step-by-step, (2) repetition, (3) tools, and (4) learning facilitators.

#### How People Prefer to Learn (Step by Step and Repetition)

The general view on the technology was clear: “It [GoLivePhone] is easy to use.” (P7, Q1), and “It [interface] has big tiles, and the overview is not cluttered.” (P4, Q2). We found step-by-step introductions, in both the course material and the number of technological functionalities offered at once, were key factors to facilitating learning: “Take it easy, step by step!” (P5, Q3). Also, frequent repetition is essential: “I see the GoLivePhone as a tool to become more knowledgeable.” (P3, Q4) but, he added, “People have to explain it to me 2–3 times.” (P3, Q5).

#### Which Tools Contribute to Learning

The smartphone community relied on one particular learning tool, which is a manual containing all course material: “If you practice using the GoLivePhone for a week and then do not use it for a month, you lose how to work with it. I am not sure I can remember everything, so that is why I need a step-by-step manual to help me out.” (P4, Q5). However, at the final evaluation, super-users initiated the request for a quick reference guide as well, of which all participants agreed: “It is difficult for people to start using the GoLivePhone. It would be handy to have a short recap for every application for daily use, to be able to look something up quickly.” (all SU, Q5).

#### Who Facilitates the Learning

Learning to use smartphone technology in a group setting was experienced as positive and motivating: “I think it is very motivating to participate with multiple people. You can exchange experiences, and you do not feel so alone.” (P4, Q5) and “I think it is a nice club. It is a little difficult though.” (P1, Q3). Furthermore, both the super-users and peers were appreciated as the relationship continued to be built: “I think it is very nice they [super-users] organized this course because I can practice the manual, challenge my difficulties and try to make it a nice thing [smartphone] for myself!” (P3, Q5) and “We get to know each other better.” (P4, Q3). A conversation between two participants in the final evaluation, shows their concerns about the appropriateness of using a phone in the presence of others. They felt technological interactions were taking over regular day-to-day interactions. P4, Q5: “I think it is necessary and valuable that super-users can give extra explanation personally in-between if you cannot keep up with the speed of the group lesson.” P3, Q5: “But people also explain things to each other on a birthday”. She goes on to explain her concern of how this is interfering. “Then there is this couple explaining things to each other, while they should celebrate a birthday! Then I think, what are you doing?”

### Personal Drivers

Personal drivers for smartphone use focusses on three different themes: motivation to use the smartphone, social motivators, and product-related values.

#### Motivations to Use the Smartphone

Within this theme, there were two prominent subthemes. Firstly, the need to prepare for the future and, for example, for health-related purposes: “I think an advantage is the tips we get from the medical applications for elderly people.” (P6, Q1). They expected that getting used to new technology might become more difficult as they aged: “Start using the GoLivePhone now, before you cannot learn it anymore.” (P7, Q3). Secondly, there was a perceived need to “Move with the times.” (P5, Q1) as to be valued as part of ongoing society: “Everything I learn helps to keep up with the modern times.” (P3, Q4) and “I think it is convenient to use a timer on the GoLivePhone because my granddaughter said an egg timer is old-fashioned.” (SU3, Q5). However, some participants explained they had limited time to practice the GoLivePhone: “There are functionalities which I cannot manage, and that is because I am swamped and have limited time to sit down and work on it.” (P4, Q5) and “I do not have time to use it, and I find it difficult, I am 86 years young.” (P1, Q3).

While the participants were motivated to respond to the calls put out by the hosts to come and learn how to use these phones, it is possible they would have responded to the call for the use of any phone, but because this had an interface designed for older adults it may have been more encouraging because they knew the technology was aimed at people like them.

#### Social Motivators

Participants are very enthusiastic because it offers connectivity to their families: “I use WhatsApp [a simple messaging service] to communicate with my grandchildren!” (P5, Q1) and “When I try to call my children, then they might not be home or do not pick up the phone. However, with WhatsApp, you are in contact immediately. I like it because I am sure I get a response, and I think they like the fact that I am not bothering them for half an hour during a phone call.” (P4, Q5). Similarly, P2 appreciates that she can keep in contact with her children: “I can see how my kids are doing, without even picking up the phone!” (P2, Q1) But she does not want the phone to replace all communication: “I use WhatsApp a lot, but I hardly make a phone call. I think WhatsApp replaces calling. However, I do not want to give a lot of personal details; I do not like that. I also do not like meeting people who are walking in the park, only looking at their phones.” (P2, Q5). Careful attention should be paid to the latter statement as a smartphone, according to her, has both positive and negative connotations.

#### Product-Related Values

Within this theme, participants gave a few examples of product-related values, as the smartphone is most commonly used for communication: “An advantage is to be able to have contact with my girlfriend. It generates more contact with people.” (P3, Q2). It is also interesting to note attitude toward the perceived usefulness of the technology toward the end of the study: “Calling and WhatsApp are the biggest advantages to me.” (P6, Q4), “There are a lot of nice things in the GoLivePhone.” (P1, Q4), “I use WhatsApp, calling, and internet the most.” (P5, Q3) and “The smartphone is indispensable for me now.” (P5, Q3). In addition, the technology gave people a feeling of safety: “It is handy to have such a phone with you.” (P1, Q2) and “I think sending messages, calling, taking pictures and having a backup in case of an emergency, are the advantages to me.” (P4, Q1).

It is interesting to note the different perceptions of the warning feature to informal caregivers. One participant stated, “I am healthy, so I do not need this feature yet.” (P4, Q5) and someone else mentioned, “They do not always need to know where I am, I think it should be possible to disable this functionality.” As the alarm functionality also shared the location, it would be interesting to see when older adults make the change from wanting to maintain their privacy to wanting to benefit by sharing information about their health with caregivers. Interestingly, a super-user's mother is using the GoLivePhone, and the super-user mentioned this location information gave a feeling of security from the caregiver perspective: “When they are away together, they are actually not alone [because she knows where her parents are in case of an emergency].” (SU1, Q5).

## Discussion

In this research, we found strategies to facilitate smartphone learning and identify the daily drivers of using this technology for aging in place. This study findings are potentially transferable to a similar context such as a small group of older adults learning new technology in a social setting and might inspire other smartphone technology research projects. The study also contributes to our general understanding of learning and using smartphone technology.

### Learning

#### How People Prefer to Learn

People made use of the two learning styles we offered: (1) practicing at home using the manual, and (2) coming to class and learning with and from peers.

##### Manual and Quick Reference Guide

Both the manual and quick reference guide were perceived as a comforting backup reference, both for learning the complete functionalities in detail (manual) and for looking things up quickly (quick reference guide). The manual used needs to match the level of expertise of the participants. Research suggests sharing notes is an ICT learning strategy when people translate the formally written manual to a more understandable and personalized style (36). Here the super-users were able to do this translation. This addresses the need that was recommended by Fondevila Gascon et al. (22) to provide clearer manuals. This highlights how the communication style most fitting this group was the translation from a company manual to an improved version, through the eyes of an older adult. So, rather than peers sharing their personalized notes, the super-user can adapt the manual before handing it out in class. Furthermore, we found it was valuable for people to be able to dedicate time for specific prioritization of different functionalities. This reflects the position of Müller et al. (21) by creating anchor points to connect technology with people's daily lives. The super-users can then suggest specific pathways for learning using the manual, but the older adult can decide which track is most meaningful for them. This promotes autonomy for the older adults, to consider their learning styles, interests, and expectations (8).

The course material consisted of an extensive text-driven binder explaining all functionalities and steps in detail. These step by step instructions are known to enable participants to learn faster and more accurately (37). In addition, the participants also requested a quick reference guide as a tool for small reminders. We created this guide focusing on specific interactions, resulting in a low-text A4 page. This addresses the needs of people who have a basic understanding already and know most steps to be executed. The quick reference guide provides security rather than being needed all the time. This guide also allows for a quick lookup of functions related to the most frequently used daily tasks. By facilitating this, we enable them to take control of their learning (38). Also, the older adults in this community associated the course material and quick reference guide as “trustworthy” and “comforting.” We observed that it is comforting for people not to have to remember everything at once in class and to have the opportunity to extend and practice to learning at home. We recommend including these tools in the learning process so that it becomes an integral part of the technology proposition itself.

##### Physical Classroom

We found needs regarding the learning process on several levels: (1) the individual (older adult), (2) the super-user (older adult, facilitator), and (3) the group (all older adults together in class). The super-users who facilitate the course need to be as motivated as others (24). Our results show general guidelines that can be followed, such as having one-on-one interaction with super-users to discuss what the focus of the next meeting should be. We also learned from our participants that the regular face to face sessions with peers made them confident learners. Seeing that others can use the technology, made participants feel they could do it as well, and so it became a joint effort in the use of new technology (10).

#### Who Facilitates the Learning

In being part of a community, people are motivated to address and work on their difficulties together. Sayago et al. (36) addressed this as collaborative and informal learning. Collaborative learning proves to be more effective for older adults than competitive or individual learning (36). In this work, we proposed two separate levels of collaborative learning: peers and super-users.

##### Peers

With peer learning, we saw the informal in-between class learning in their natural social environment (24), where people help each other, so everybody learned at the same pace. They all have the same goal to get acquainted with technology, as the technology has been unfamiliar from the start for all of them, together they make faster progress in learning.

##### Super-Users

In addition to peers, super-users were the people who hosted the session, who took the lead in facilitating which steps to practice next and joined in executing tasks together. Master-apprentice roles is an acknowledged way of learning (39), that transfers to this context, to make this work trust in each other is essential. The availability of support, in this case through super-users, influences how older adults experience certain challenges (10). And sometimes super-users changed roles between facilitating and being a peer learner, as they relearn and repeat steps with their peers one on one.

##### Sustainable Learning Process

The compelling aspect of this collaborative learning community is that peers can grow toward becoming super-users, which turns this approach into a sustainable learning process in the community. We have seen 1 year after this project, there have been four different groups practicing the smartphone, and from this study, everyone became a super-user later. It is a low-cost way to facilitate teaching, and the social value of getting together to learn with peers is an essential motivator. We believe this role of super-user stimulates continued learning, as people seem to value being recognized as a super-user (40). This credit gives an extra stimulus for participants to become super-users.

##### Acknowledgment and Support From the Municipality

We have seen this growing group of older adults to come together and learn has caught attention from the municipality as they benefit from a healthier and happier community. Therefore, the municipality subsequently subsidizes the ongoing service costs of the smartphone for all participants who accomplished the first class. This need for organizational collaboration is expressed by policy advisors in order to enable successful implementations of technology for aging in place (41). Furthermore, participants of the smartphone classes gained recognition as they were acknowledged in a local news article and received a certificate of their successful participation.

##### Informal Atmosphere

We saw a social atmosphere where people shared personal learning stories. Work from Sayago et al. (36) shows such learning does not depend on knowing more or less as your peers, but the social and informal atmosphere itself is motivating. We saw through this informal atmosphere, that accepting new functionalities was easier, as users saw their peers using this. However, there is a limit to this informal setting, for two participants a birthday gathering was not appropriate for example. This shows, on the one hand, the integration of the device in people's daily life but, on the other hand, some non-acceptance (yet) of others. We believe the learning atmosphere should be informal, but the importance of attending classes and of making use of fixed timeslots to learn together needs to be emphasized. We have seen our participants had a busy lifestyle, we observed people needed frequent repetition. By having a dedicated timeslot to learn, they could keep up with the pace.

### Personal Drivers

Within the category of personal drivers concerning smartphone use, we will elaborate on three different themes: motivations to use the smartphone, social motivators, and product-related values.

#### Motivations to Use the Smartphone

##### Preparing for the Future and Not for Me (yet)

Participants indicated one reason for joining the class is preparing for the future, when they might be more dependent. This illustrated how the participants were engaged in future thinking (42). This need is prevention-driven, to prepare for the changes which might follow in later life when more support is needed. Most participants saw the smartphone as a system, which could help them to achieve that and provide a feeling of being prepared. Not only did they think about the use of a specific application for today or tomorrow, but the driver for some of our participants was also to get acquainted with the smartphone before they could not learn it because, for example, the onset of dementia. They saw the smartphone as a means of giving them a secure, safe, and in control perspective on the future. In addition to keeping up with modern times, as reflected in the findings of Rosales et al. (43).

We found our participants were still healthy and not in need of the health support functions of the smartphone technology yet. Literature shows that older adults perceive certain stigmas with technology designed for them, such as is discussed in the work of Neven (44) where participants imagined potential users of a health robot as a lonely person who is in need of care and company. However, our participants mentioned that it motivated them to start using the smartphone, and getting acquainted with the novel technology now, and be able to start integrating the device into their daily lives. This makes sense for older adults who want a device that addresses their current needs and to use a technology shaped in dialogue with their everyday practice now (45), with options to support them in a different way later with regard to their personal health. As was shown by their wanting to move with the times, and not be left out (20), our participants happily agreed to learn a smartphone now 'with some additional care functions for later'.

##### Fun and Social Functionalities

Often technology focusses on what is no longer possible, trying to “solve aging problems” (46). However, we saw that the value technology brings is much more than that. It creates opportunities to enrich people's daily life. For example, it is an easy way to stay in contact when living far away from each other. Therefore, we have to recognize and emphasize the need for fun and social smartphone functionalities (such as WhatsApp) in addition to care functionalities (such as fall prevention). These do not have to be contradictory or independent from each other (9). People might not feel like they need care services but instead want to interact and share meaningful things with their surrounding network (47). These drivers can be used to fuel learning and link a technology to different essential real-life needs (36), which can be complementary to daily life now as well as in the future.

#### Social Motivators

##### Emotional Response to Technology

Sayago et al. (36) suggest learning is driven by real-life situations, such as a son who keeps telling his parents to learn to use email for communication. Children could for example lay a major role in motivating technology addition as suggested by Fausset et al. (48). And even if the older adults themselves do not believe it is important, if family members think it is important, they may still comply with them (49). Our study showed, in the communication and use of WhatsApp, that the smartphone technology facilitated participants to stay in touch with social networks. These findings expand on existing literature showing that in addition to showing a willingness to use technology, it is crucial to building the experience toward not only a functional response but an emotional one such as facilitated by social contact (10).

##### Immediate and Flexible Contact

As people value the smartphone as an enabler to have contact with their loved ones (50), they also specifically point out the value of immediate and flexible contact. Our participants compare sending a message vs. a phone call and prefer the message so that their busier family members can respond any time rather, and they do not feel like they are bothering them with a long call. This extends the findings of Lindley et al. (51), saying that older adults do not want to become burdensome or intrusive when staying in contact.

#### Product-Related Values

##### Security and Privacy

While we see, in general, a positive view of people expressing why they value the smartphone, the security and privacy topic still evoked mixed responses among the participants. On the one hand, our participants suggested they feel safer because in our system they could chose an informal caregiver to reach out to them and monitoring their location, whenever in need of help. On the other hand, participants mentioned they value their privacy and do not want to be tracked by anyone else (52). This is a personal preference, and in some cases, it is the older adult and, in some cases, it is the (informal) caregiver who might feel safer due to the technology. With our smartphone, older adults can decide with whom they share information, which is important for data privacy (53). There we propose that the freedom of choice should always be facilitated by technology, also in the case of people in need.

## Conclusions

In this study, with seven users and four super-users, we have explored a social learning environment for older adults to learn how to use new technology and share their knowledge. Older adults in this study prefer to take a step by step approach, with the support of their peers and a plain-language manual. They showed to be motivated to learn to keep in touch with friends and family. Additionally, super-users contribute to a sustainable learning process as users could later become super-users and can help understand other older adults to use technology. This means people setting up learning experiences for older adults should consider peer to peer and user lead approaches.

These findings are of an explorative nature and therefore not generalizable to a broader population of older adults, we suggest that our findings are transferable to similar groups and could inspire other researchers working with individuals in a specific context. Furthermore, we have addressed some touchpoints that can support new technology learning and adoption, depending on people's previous technology experience and current context in which they are learning. Currently, we worked together with a group of people who did not have previous smartphone experience. However, for an increasing number of older adults, smartphones will become a part of their lives. Thus, when designing for this target group, it is also important to facilitate a stimulating and social learning experience.

## Data Availability Statement

The raw data supporting the conclusions of this article will be made available by the authors, without undue reservation.

## Ethics Statement

Ethical review and approval was not required for the study on human participants in accordance with the local legislation and institutional requirements. The patients/participants provided their written informed consent to participate in this study.

## Author Contributions

MH and RB conducted the fieldwork. MH took the lead in writing the manuscript. All authors discussed the results and contributed to the final manuscript.

## Conflict of Interest

The authors declare that the research was conducted in the absence of any commercial or financial relationships that could be construed as a potential conflict of interest.
